# Accumulation of Astaxanthin by a New *Haematococcus pluvialis* Strain BM1 from the White Sea Coastal Rocks (Russia)

**DOI:** 10.3390/md12084504

**Published:** 2014-08-15

**Authors:** Konstantin Chekanov, Elena Lobakova, Irina Selyakh, Larisa Semenova, Roman Sidorov, Alexei Solovchenko

**Affiliations:** 1 Biological Faculty of Lomonosov Moscow State University, 1/12 Leninskie Gori, Moscow 119234, Russia; E-Mails: chekanov@mail.bio.msu.ru (K.C.); elena.lobakova@mail.ru (E.L.); i-savelyev@mail.ru (I.S.), semelar@mail.ru (L.S.); 2 Timiryazev Institute of Plant Physiology, Russian Academy of Sciences, 35, Botanicheskaya str., Moscow 127276, Russia; E-Mail: roman.sidorov@mail.ru

**Keywords:** astaxanthin, carotenogenesis, fatty acids, green microalgae

## Abstract

We report on a novel arctic strain BM1 of a carotenogenic chlorophyte from a coastal habitat with harsh environmental conditions (wide variations in solar irradiance, temperature, salinity and nutrient availability) identified as *Haematococcus pluvialis* Flotow. Increased (25‰) salinity exerted no adverse effect on the growth of the green BM1 cells. Under stressful conditions (high light, nitrogen and phosphorus deprivation), green vegetative cells of *H. pluvialis* BM1 grown in BG11 medium formed non-motile palmelloid cells and, eventually, hematocysts capable of a massive accumulation of the keto-carotenoid astaxanthin with a high nutraceutical and therapeutic potential. Routinely, astaxanthin was accumulated at the level of 4% of the cell dry weight (DW), reaching, under prolonged stress, 5.5% DW. Astaxanthin was predominantly accumulated in the form of mono- and diesters of fatty acids from C16 and C18 families. The palmelloids and hematocysts were characterized by the formation of red-colored cytoplasmic lipid droplets, increasingly large in size and number. The lipid droplets tended to merge and occupied almost the entire volume of the cell at the advanced stages of stress-induced carotenogenesis. The potential application of the new strain for the production of astaxanthin is discussed in comparison with the *H. pluvialis* strains currently employed in microalgal biotechnology.

## 1. Introduction

The red ketocarotenoid Astaxanthin (Ast) is a potent antioxidant exerting a plethora of health-beneficial effects in human and animal organisms. It is of high demand as an ingredient of cosmetic, medical and dietary formulations [1,2] as well as quality feed for aquaculture. In particular, the red color of the crustacean shells and salmon meat is due to the presence of Ast; the only source of Ast in animals is their diet [3]. At present, most of the feed Ast is chemically synthesized although the synthetic pigment, unlike natural Ast, is a racemic mixture containing a substantial proportion of the stereoisomers lacking the biological activity [3].

The richest natural source of Ast is the chlorophyte *Haematococcus pluvialis* Flotow [4] that accumulates the pigment in an amount of up to 3%–6% of cell dry weight (DW) under unfavorable environmental conditions [5]. Essentially a freshwater alga, *H. pluvialis* survives in small rain pools under extremely volatile conditions such as extreme temperatures, low nutrient availability and solar irradiance [6] mainly in form of Ast-rich non-motile coccoid cells with an exceptional tolerance of the adverse conditions [7,8,9,10]. The massive accumulation of Ast in *H. pluvialis* depends on and is accompanied by the enhanced biosynthesis of neutral lipids, mainly triacylglycerols (TAG) [11]. The reason for this is that Ast is deposited in cytoplasmic lipid droplets (LD) comprised by TAG, mainly in the form of FA esters. Accordingly, *H. pluvialis* can also be a source of valuable FA e.g., oleic acid [5,12].

In spite of its high bioavailability and numerous beneficial effects, natural Ast from microalgae hardly can compete with its synthetic analog due to high production cost and limited productivity of the commercial *H. pluvialis* strains [2,3]. Obviously, at least a two-fold increase in the Ast productivity of current strains (which is at the level of ca. 3% DW) is necessary for natural Ast to outcompete the synthetic pigment [13]. Moreover, mass cultivation of *H. pluvialis* is highly demanding of fresh water, which may not be available at the site of cultivation. Therefore bioprospecting of more stress-tolerant *H. pluvialis* strains is important to reduce the costs of the Ast-enriched biomass production e.g., by the use of brackish water. We paid close attention to White Sea coastal rocks characterized by a particularly harsh environment expecting to obtain microalgal isolates naturally adapted to the adverse conditions. In the present work, we obtained a detailed characteristic of a novel *H. pluvialis* strain from an arctic sea and estimated its suitability for Ast production.

## 2. Results and Discussion

### 2.1. The Habitat of the New Strain

**Figure 1:**
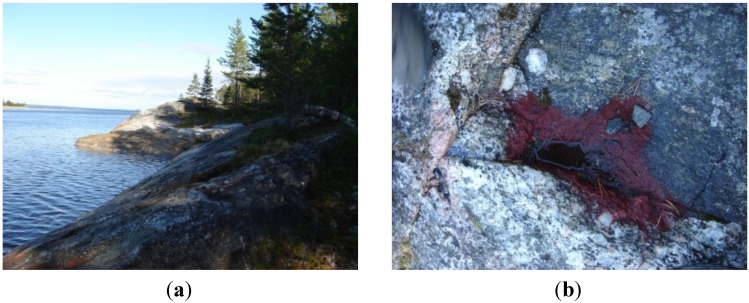
(**a**) Coastal rocks at Kost’yan Island, White Sea; (**b**) The red crust formed by the astaxanthin-rich hematocysts in a drying rock bath.

The carotenogenic microalga designated as BM1 described in this paper was originally discovered as reddish crusts on the northern slope of black granite-gneiss coastal rocks on Kost’yan island (66°29′47″ N, 33°24′22″ E), White Sea (Figure 1). This habitat is characterized by harsh environmental conditions even during the warm season. Thus, during polar days (March to August), the northern slopes of the cliffs are constantly illuminated by sun. As a result, the water filling the rock baths inhabited by the microalga is considerably warmer than the seawater (Supplementary Table S1). Sharp fluctuations of salinity are also typical of this habitat due to enhanced evaporation from sun-heated rocks, especially during windy weather, and regular inflow of seawater from high tides or fresh water from rain and melting snow.

**Figure 2:**
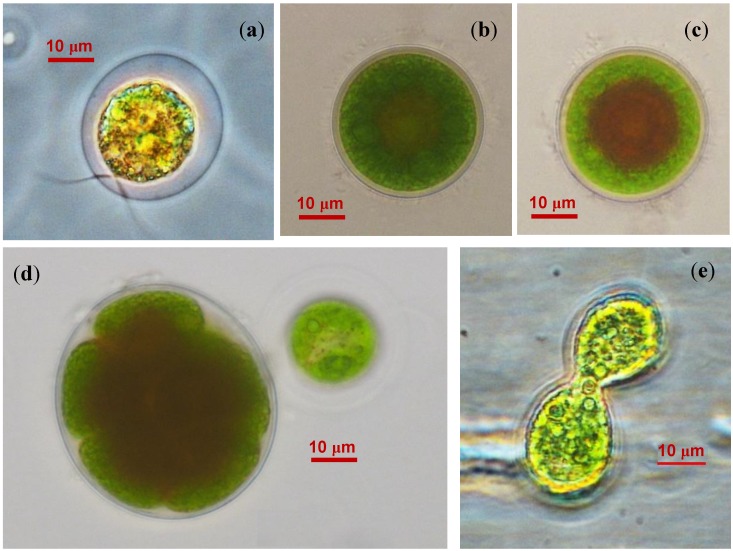
Life cycle stages of BM1 isolate (**a**) Zoospore; (**b**) Coccoid green cell; (**c**) Coccoid green cell with red-colored lipid droplets; (**d**) Sporangium; (**e**) Putative isogamous sexual process.

Until the end of June, the microalga dwelled in the baths mainly as motile biflagellate green zoospores or coccoid non-motile cells (Figure 2b). In July, the microalga was represented mainly by large (up to 80 μm in diameter) bright red-colored coccoid resting cells (Supplementary Figure S2a) referred to below as “red” cells. By the end of July, the baths usually dried out and the “cells” formed the reddish crusts on the rock surface (Figure 1). In addition to the microalgal cells, thin (1–3 μm in diameter) filamentous heterocyst-lacking cyanobacteria (III subsection, presumably Oscillatoriaceae) were encountered in the samples. The surface of the “red” cells was often decorated by numerous bacterial rod-like cells attached by their apical ends (not shown).

To the best of our knowledge, the literature on isolation of *H. pluvialis* from arctic regions is scarce and limited to the isolates from freshwater habitats. A cold-tolerant strain of *H. pluvialis* capable of growth at a low (4–10 °C) temperature was recently isolated from a freshwater lagoon in Blomstrandhalvøya Island (Svalbard) [14]. In contrast to BM1, this strain was incapable of sustained growth at temperatures higher than 15 °C since these conditions triggered the formation of the red-colored cysts in the latter. Notably, the enrichment culture of the freshwater arctic strain also contained filamentous heterocyst-lacking cyanobacteria from the III subsection.

### 2.2. The Cell Morphology and Ultrastructure

Several morphological types of cells were found in BM1 cultures. The first cell type was represented by motile spherical zoospores (15–20 μm in diameter with a mucous sheath) with two isokont anterior flagellae and a discoid eyespot near the cytoplasmic membrane at the cell anterior (Figure 2a). The zoospores featured a cup-shaped chloroplast occupying almost the entire cell volume. Another type was comprised by non-motile coccoid cells (20–40 μm in diameter; Figure 2b). The cells contained a centrally located spherical nucleus. Palmelloid clusters of two to eight cells were formed occasionally. Under adverse environmental conditions, the coccoid cells increased in size (up to 80 μm) and accumulated red-colored spherical inclusions in the cytoplasm, which tended to cluster around the nucleus (Figure 2c). The red inclusions gradually occupied the entire cytoplasm volume resulting in the formation of resting “red” cells (Supplementary Figure S2a).

The isolated microalgae propagated mainly via asexual reproduction forming sporangia containing two to eight autospores (Figure 2d). After shedding the sporangium wall, the newly formed cells remained attached to each other for a long time; as a result, the culture tended to accumulate four-cell clusters. Under non-optimal cultivation conditions, e.g., in a highly diluted culture, small, pear-shaped fast-moving biflagellate cells similar to gametes as described by Triki *et al.* [15] were encountered. Occasionally, these cells underwent conjugation resembling isogamous sexual process (Figure 2e). Dead cells with transparent content were also present in the culture (not shown). One could conclude that the life cycle and cell morphology recorded in the BM1 isolate as well as the ability to accumulate the red pigment in the “red” cells are consistent with the characteristic traits of *Haematococcus pluvialis* Flotow [15,16,17].

**Figure 3:**
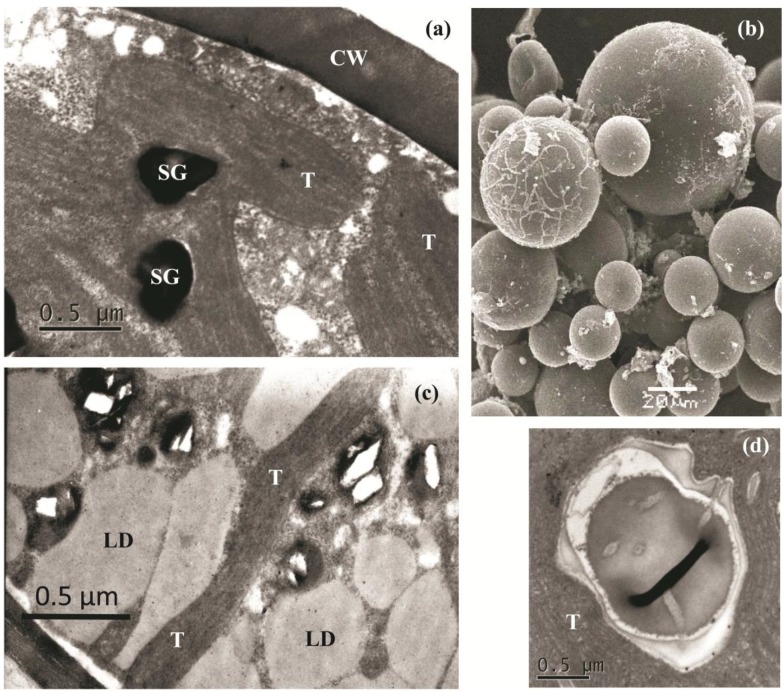
Electron micrographs of *H. pluvialis* BM1: (**a**) transmission electron microscopy (TEM) of a “green” cell; (**b**) scanning electron microscopy (SEM) of enrichment culture comprised of different cell types; (**c**) TEM of a “red” cell; (**d**) Pyrenoid structure typical of BM1 cells. CW—cell wall; LD—lipid droplets; SG—starch grains; T—thylakoids. Note the absence of LD in the “green” cells (**a**) and their presence in the “red” cells (**c**).

To characterize the newly isolated microalga, we investigated its cell ultrastructure. It should be noted that ultrastructural studies of *H. pluvialis* are generally more difficult in comparison to most of green microalgae, mainly due to the presence of tough cell walls complicating the chemical fixation, embedding and preparation of ultrathin sections [18]. Indeed, we found that the thick cell wall of BM1 was, like that of *H. pluvialis* aplanospores, extremely resistant to mechanical disruption and chemical agents and presented difficulties for electron microscopy. Nevertheless, both transmission (Figure 3a,c,d) and scanning (Figure 3b) electron micrographs of “green” and “red” BM1 cells were obtained.

As was shown by electron microscopy, the BM1 cells at different stages of life cycle were spherical and 18–59 μm in diameter (Figure 3b). Green flagellated and palmelloid cells possessed a thick (0.64–0.8 μm) extracellular matrix, which was thinner in the palmelloid and “red” cells. All kinds of palmelloid cells formed (up to 0.4 μm) the thick cell wall. The chloroplast contained two to ten pyrenoids and a few starch grains (Figure 3a,d). In the resting “red” cells, a pronounced decrease in the thylakoid volume and number was recorded; large merging lipid droplets were also present, which (Figure 3c) eventually occupied almost the entire cell volume.

### 2.3. Molecular Identification

**Figure 4:**
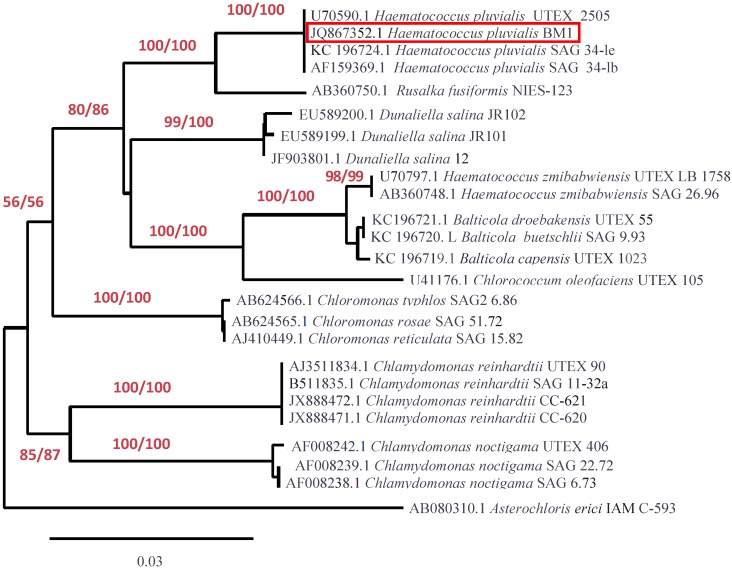
Phylogenetic relationships of BM1 isolate as revealed by 18S rRNA gene sequence. The optimal tree is shown. The percentage of replicate trees in which the associated taxa clustered together in the bootstrap test (1000 replicates) are shown under the branches for maximum likelihood/neighboring-joining (ML/NJ) method, respectively. All positions containing gaps and missing data were eliminated from the dataset. There were a total of 782 positions and 25 taxa in the final dataset. Phylogeny analysis was conducted in PhyML 3.0 and BioNJ. The tree was rendered using TreeDyn 198.3 software (GEMI Bioinformatics, Montpellier, France).

In order to reveal the taxonomic identity of the BM1 isolate we obtained a partial sequence of its 18S rRNA gene (GenBank ID JQ867352.1). The homology search using the Basic Logical Alignment Search Tool (BLAST) showed the maximum (99%–100%) identity of the sequence with respective sequences of a number of known *H. pluvialis* strains (Figure 4). The phylogenetic analysis of the BM1 isolate showed that it belongs to *Haematococcus pluvialis* Flotow, the single species in the genus *Haematococcus* nested in the Chlorogonium clade, Volvocales, Chlorophyceae which is consistent with the generally accepted results of molecular identification of *H. pluvialis* [19]. Basing on the findings described above, the isolate BM1 was designated as *H. pluvialis* BM1.

### 2.4. Growth and Carotenogenesis

#### 2.4.1. Biomass Accumulation and Pigment Composition

In order to evaluate the potential of *H. pluvialis* BM1 for biotechnology the isolate was cultivated in a closed bubble-column photobioreactor as described in the “Experimental” Section (see also Supplementary Figure S2d). The “green” cell cultures reached a maximum cell density of 1.6 × 10^6^ mL^−1^ (37 mg·mL^−1^ Chl, 6 mg·mL^−1^ Car, ca. 1.0 g·L^−1^ of cell dry weight, DW) in 5–7 days corresponding to the specific growth rate, μ = 0.095 day^−1^ at the exponential phase (Supplementary Figure S2). At the exponential growth phase, the culture was comprised, to a considerable extent, by dividing “green” cells (Figure 2). The cell suspension was bright green in color due to low (ca. 0.18 ± 0.01) Car/Chl ratio (by weight). The Car at this growth phase were represented exclusively by primary carotenes and xanthophylls, there was no detectable presence of Ast (see Section 2.4.3 below and Figure 6). After 10 day of cultivation, accumulation of astaxanthin was detected and the Car/Chl ratio gradually increased, apparently, due to nitrate depletion in the medium.

#### 2.4.2. Stress-Induced Astaxanthin Accumulation

To induce the massive accumulation of Car, the “green” cells of *H. pluvialis* BM1 were transferred to the stressful conditions mimicking, to a certain extent, the nutrient deficiency and excessive solar irradiation in their natural habitat. Specifically, the cells were washed, resuspended in distilled water, and exposed to irradiance one order of magnitude higher than that optimal to the “green cells” (see the “Experimental” section). Under these conditions, most of the cells displayed a rapid induction of Ast biosynthesis and turned into non-motile “red” cells (Supplementary Figure S2a).

The induction of carotenogenesis occurred in the background of a sharp decline of Chl content. As a result, the shape of the absorption spectra of extracts from the “red” cells was governed by Ast absorption (Supplementary Figure S2b). Notably, the cells of *H. pluvialis* BM1 even after prolonged stress exposure always retained a certain amount of Chl; only dead colorless cells possessed no detectable Chl content. On the whole, the dynamics of stress-induced Car accumulation displayed by BM1 was similar to that recorded in known *H. pluvialis* strains [20].

High performance liquid chromatography (HPLC) analysis (see Section 2.4.4 below) confirmed that nearly 99% of total Car in the “red” cells were represented by Ast reaching 5.0%–5.5% DW by the 6th day of stress (corresponding to a Car/Chl of 13.0 ± 0.1). After the 6th day of stress exposition, the Ast content declined sharply (Supplementary Figure S2c). This process was manifested by a massive appearance of bleached cells.

#### 2.4.3. Salinity Effects on the Growth of the “Green Cells”

The abrupt changes of salinity characteristic of the habitat of BM1 (see Section 2.1) suggest an increased ability to acclimate to this factor in the microalga under investigation. To obtain a preliminary estimation of BM1 salinity tolerance, we cultivated the microalga under salinity similar to that of the rock bath water (25‰), which is typically below the White Sea water salinity (29‰) because of dilution with rainwater.

**Figure 5:**
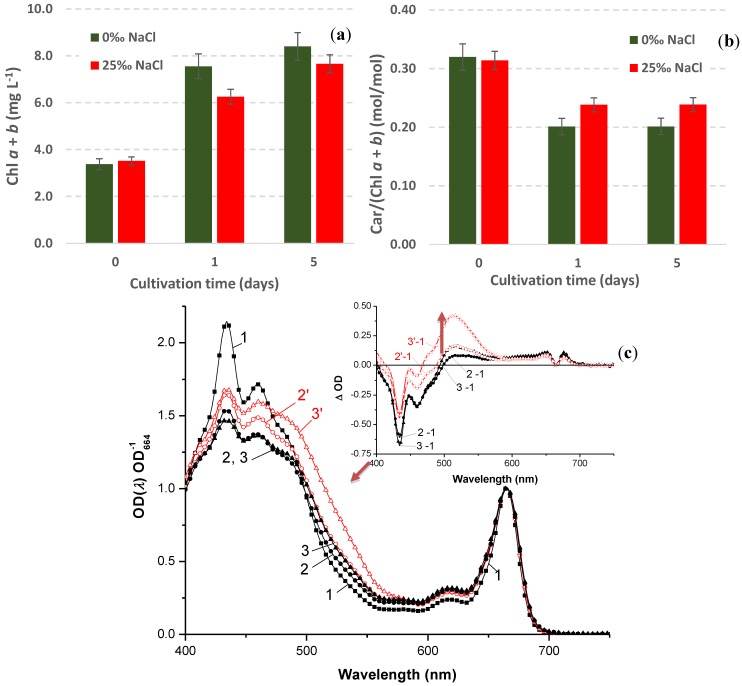
Effects of 25‰ NaCl on (**a**) chlorophyll accumulation; (**b**) carotenoid-to-chlorophyll ratio; and (**c**) normalized absorption spectra of the cell dimethyl sulfoxide (DMSO) extracts of *H. pluvialis* BM1 “green” cell culture. The spectra for (*1*) initial culture (Day 0) as well as those recorded after one day (*2*, *2*′) and five days (*3*, *3*′) of cultivation in the medium containing (*2*′, *3*′) or lacking (*2*, *3*) NaCl are shown. Insert: different absorbance spectra of the extract spectra presented in the panel (**c**). Note a positive peak in the green region indicative of a limited accumulation of astaxanthin by the fifth day of cultivation in the presence of NaCl.

It was found that the increase in salinity *per se* did not trigger a decline in Chl accumulation by the culture (Figure 5a) or an increase in Car accumulation over Chl (Figure 5b) typical of the stress-induced carotenogenic response. During first 5–7 days, the kinetics of growth on the saline medium did not differ significantly from that on the medium lacking NaCl (see Section 2.4.1 above and Supplementary Figure S1). Only a limited accumulation of Ast was detected in the cultures grown at 25‰ NaCl (insert in Figure 5c).

**Figure 6:**
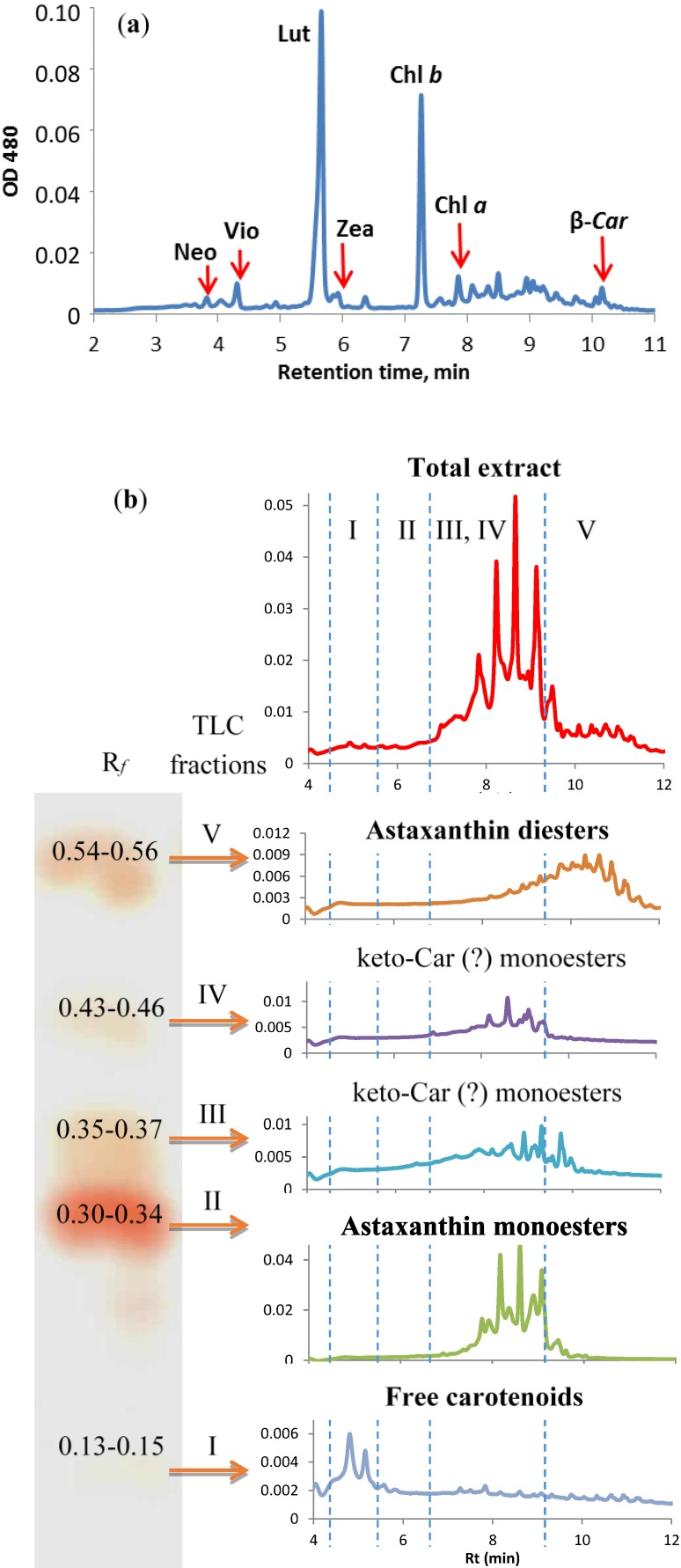
Pigment composition of *H. pluvialis* BM1 cells at different cultivation stages (**a**) The “green” cells (high performance liquid chromatography (HPLC)); (**b**) The “red” cells (thin-layer chromatography (TLC) + HPLC).

At the same time, it is known that the addition of 0.8% NaCl (8‰) to the medium normally causes a cessation of growth of *H. pluvialis* [7,20]. In view of these facts, the new strain *H. pluvialis* BM1 seems to have a higher tolerance to salinity stress although a more detailed investigation of the limits and possible side effects of its salinity tolerance is necessary. Nevertheless, this finding may be important for the biotechnology of Ast production in the areas with a limited supply of fresh water suggesting the possibility of cultivation of “green” cells of *H. pluvialis* BM1 in brackish water.

#### 2.4.4. Stress-Induced Changes in Pigment and Fatty Acid Composition

Under conditions conducive to rapid growth of the culture, the pigment composition of *H. pluvialis* BM1 was typical of green algae thylakoid membranes [21] including Chl *a* and *b* as well as primary Car; only trace quantities of Ast esters were detected (Figure 6a).

Thin-layer chromatographical (TLC) separation of the extracts from the “red” cells yielded five pigment fractions (Fractions I–V, Figure 6b). The absorption spectra of all fractions resembled those of pure Ast (*λ*_max_ = 490). Incubation in air at room temperature for 10–15 min resulted in the long-wave shift of the maximum to 494 nm typical for astacene, an oxidation product of Ast [22]. The fast conversion of the pigment from the Fraction I to astacene suggests that the Fraction I contained non-esterified (free) Ast. The bulk (ca. 70%) of the Ast in the “red” cells was found in the Fractions II (*R*_f_ = 0.30–0.34) and III (*R*_f_ = 0.35–0.37). The Fractions IV (*R*_f_ = 0.43–0.46) and V (*R*_f_ = 0.54–0.56) were substantially less abundant. Free Ast (the Fraction I) comprised less than 3.5% of the total Ast.

**Table 1:** Fatty acid composition (*mas.-%*) of esterified carotenoid fractions of *H. pluvialis* BM1 “red” cells.

Fatty Acid	TLC Fraction			
	II ^(a)^	III ^(b)^	IV ^(c)^	V ^(d)^
14:0	7.8	4.9	5.0	-
16:0	**41.1**	**55.3**	**50.4**	-
7–16:1	5.9	6.5	4.4	2.3
7,10–16:2	-^*^	-	-	**12.0**
18:0	5.7	7.9	6.7	1.7
9–18:1	**16.3**	**17.3**	**17.7**	**23.8**
11–18:1	1.1	-	-	1.3
9,12–18:2	**10.2**	**7.4**	**9.4**	**15.8**
6,9,12–18:3	1.0	-	-	0.8
9,12,15–18:3	5.9	-	-	5.0
13–22:1	2.2	-	4.1	0.5
UI ^**^	**0.694**	**0.387**	**0.450**	**1.156**

^*^ not detected; ^**^ unsaturation index ^(a)^ 6,9,12,15–18:4, 20:0, 22:0, 24:0 also were present; the concentration of each was 0.6%–0.8%; ^(b)^ also contained 0.5% of 20:0 FA; ^(c)^ 12:0, 20:0 and 22:0 were present, the concentration each was 0.7%–0.9%; ^(d)^ also contained 7,10,13–16:3—1.9%, 4,7,10,13–16:3—2.3% and 20:0, 22:0, 24:0, the concentration of each was 0.1%–0.2%.

The HPLC-diode-array detector (DAD) analysis of the Fraction I as obtained by TLC confirmed the presence of free Ast. The HPLC elution profile of the Fractions II and III pigments contained eight major peaks (*R*_t_ 8–10 min). One could suggest that these peaks correspond to individual molecular species of Ast monoesters. The Fraction V harbored a large number of nonpolar compounds containing Ast chromophore, most probably Ast fatty acid diesters.

Notably, accumulation of Ast in *H. pluvialis* BM1 cells took place along with a significant increase in neutral lipids in cytoplasmic lipid droplets as evidenced by vital staining of the “red” cells with Nile Red. The gas chromatography-mass spectrometry (GC-MS) fatty acid analysis (Table 1) demonstrated that the fatty acid profile of the Ast monoesters was dominated by palmitic (16:0), oleic (Δ9–18:1), and linoleic (Δ9,12–18:2) acids; it was similar to that of the known *H. pluvialis* strains [11,12,23]. Interestingly, The FA from the diester fraction (V) possessed a nearly two-fold higher unsaturation index in comparison with those from the monoester fractions (II–IV). Taking into account the strong differences in FA composition of the mono- and diesters of Ast in *H. pluvialis* BM1, one may speculate that (i) their FA originate from different pools and (ii) the enzymes responsible for biosynthesis of the different classes of Ast esters possess a different substrate specificity.

## 3. Experimental Section

### 3.1. BM1 Isolation and Obtaining of Its Culture

Samples were collected from red-colored bath tiles filled with semi-saline water found in the supralittoral zone of the Kost’yan Island (66°29′47″ N, 33°24′22″ E) in the White Sea. Water samples and red plaque (Figure 1b) scrapings from the surface of parched “bath-tile” mud cracks were collected in June–August of 2011 and 2012. The samples were sealed in sterile polypropylene bags. Within three hours, the precipitate of the water samples was transferred to two mL of BG-11 medium [24]. The plaque scrapings were transferred in five mL of BG-11 in sterile 15 mL glass vials. Samples were incubated in 15 mL sterile glass vials at 20 °C under daylight for one month. Algological pure cultures were obtained at Pertsov White Sea Biological Station of Moscow State University. Single red-colored cells and sporangia were placed in five mL of BG11 in sterile tubes using a micromanipulator and incubated under a white fluorescent lamp (40 μE·m^−2^·s^−1^) at room temperature for one month. The resulting cultures were inoculated onto Petri dishes with solidified BG11 medium (containing 2% agarose) using a dilution method and incubated under the same conditions until green-colored colonies became apparent (2–3 weeks). The colonies were transferred to the tubes containing sterile BG-11 medium and incubated under the same conditions.

### 3.2. Culture Handling and Maintaining

The “green cells” of the microalgal isolate BM1 were cultivated in 400 mL of BG11 medium in 600-mL glass columns (6.6 cm internal diameter, 1.5 L·volume). The cultures were grown under continuous photosynthetically active radiation (PAR) illumination of moderate (40 μE·m^−2^·s^−1^) intensity as measured with a LiCor 850 quantum sensor (LiCor, Lincoln, NE, USA) in the center of an empty column by white light emitting diode source. For the induction of carotenogenesis, the cells were incubated in distilled water at high (480 μE·m^−2^·s^−1^) PAR intensity. The cultures were continuously bubbled with air (1 v·v^−1^·min^−1^); the temperature was maintained at 27 °С.

To obtain a preliminary estimation of salinity tolerance of BM1, the cells were cultured in 250-mL flasks containing 80 mL of BG-11 medium supplemented with 25 g·L^−1^ NaCl (BG-11 lacking NaCl was used as the control) in a shaker incubator at 100 RPM, 27 °C and 40 μE·m^−2^·s^−1^ PAR.

Cell dry weight (DW) was measured according to [20]. Cells were counted in a hemocytometer.

### 3.3. Molecular Identification

To destroy the tough cell wall, the 1.5-mg cell samples collected for DNA extraction were subjected to three cycles of freezing in liquid nitrogen and subsequent thawing at room temperature. The samples were incubated for one hour in 300 μL of citrate-phosphate buffer (pH 5.0) containing 10 mg·mL^−1^ cellulase (Fermentas, Vilnius, Lithuania), 10 mg·mL^−1^ pectinase (Fermentas, Vilnius, Lithuania) and 1mM EDTA at 37 °C for one hour. The samples were incubated with 2% sodium dodecyl sulfate for one hour at 40 °C. Next 400 μL of 1M NaCl were added and allowed to stand overnight for protein salting. Then standard phenol-chloroform extraction was performed [25]. The DNA sample purity was evaluated by electrophoresis in 1.5% agarose gel with ethidium bromide.

For amplification of the 18S rRNA gene fragment, the following primers were designed using Gene Runner 4.0.9.68: 5′-tggctcattaaatcagttatag-3′, 5′-ccaagaatttcacctctgaca-3′. Polymerase chain reaction (PCR) was performed on a Bio-Rad DNA engine (PTC 200, Hercules, CA, USA) using the amplification profile of 94 °C for 3 min initial denaturation, 94 °C for 20 s, 60 °C for 25 s, 72 °C for 35 s, 30 cycles; final elongation—72 °C for 5 min. The PCR mixture contained 10 ng of the algae genomic DNA in 25 μL of 1× PCR Buffer for Tersus (Evrogen, Moscow, Russia) containing 200 μM of each dNTP, 0.2 μM of each primer and 0.5 μL of 50× Tersus Taq polymerase (Evrogen, Moscow, Russia). The PCR products were purified using a Cleanup Standard PCR purification kit (Evrogen, Moscow, Russia) and sequenced on ABI Prizm 3730 (Applied Biosystems, Life Technologies, Grand Island, NY, USA) in both directions.

Sequences were searched against the NCBI GeneBank (nucleotide collection nr/nt database) using BLAST. For the data analysis, 24 sequences from GenBank were selected. Multiple alignment of the sequences were conducted using ClustalW2 online tool [26]. There were a total of 782 positions and 25 taxa in the final dataset. The following parameters of pair-wise alignment were used Alignment type: slow, DNA weight matrix: IUB, gap open: 100, gap extinction: 10.0. The parameters of multiple alignments are given below. DNA weight matrix: IUB, gap open: 100, gap extinction: 10.0, gap distances: 10, no end gaps, iteration type: “tree”, number of iterations: 10. Phylogenetic trees for multiple alignments were designed with using maximum likelihood (ML) method [27] in PhyML 3.0 [28] and neighbor-joining method (NJ) [29] in BioNJ [30,31].The best of nearest neighbor interchange (NNI) and subtree pruning and regrafting (SPR) were used for tree improvement in PhyML 3.0. HKY85 model of DNA evolution [32] were used. For other parameters of the analysis, the default values were left. Trees were rendered using program TreeDyn 198.3 [33]. The accuracy of the tree topology was tested using bootstrap analysis [34] with 1000 replicates.

### 3.4. Pigment Analysis

Routinely, the 0.5–1.0 mL aliquots of cells suspension were extracted with dimethyl sulfoxide (DMSO). The cells were pelleted by centrifugation (5 min, 12,000 rpm), the supernatant was removed and the cells were incubated with 1.5 mL of DMSO at 75 °C for 15 min, and the cells were removed by centrifugation; then the extraction was repeated. Chlorophyll (Chl) *a*, Chl *b* and total carotenoids (Car) in the extract were assayed spectrophotometrically [35] with an Agilent Cary 300 (Agilent, Santa Clara, CA, USA) spectrophotometer in standard 1-cm cuvettes.

For the fatty acid (FA) and pigment assay, the extraction by Folch was used [36]. In this case Chl *a*, *b,* and total Car contents were determined in lower (chloroform) fraction [37]. The pigments were initially separated by TLC on silica gel plates (Sulifol, Kavalier, Prague, Czech Republic). The hexane:benzene:chloroform (5:5:2, by volume) and hexane:chloroform:benzene (10:20:1, by vol.) were used for separation of “green” and “red” cell extracts, respectively. The spots obtained after separation of the “red” cell extracts were gently scrapped from the plate by scalpel and eluted with chloroform.

Both the total pigment extracts and eluted fractions were subjected to HPLC analysis using an Alliance 2995 separation module (Waters, Milford, MD, USA) equipped with a 150 × 4.5-mm Prontosil RP C-18 column (Knauer, Berlin, Germany) maintained at 25°C and a Waters e2695 DAD detector (Waters, Milford, MD, USA). The gradient elution of pigments was achieved at a flow rate of 1 mL·min^−1^ using (A) acetonitrile, (B) water, and (C) ethyl acetate mixtures (vol. %): for the “red” cell extracts, 98:2:0 (2 min), 40:0:60 (10 min), 0:0:100 C (2 min) followed by 6-min re-equilibration of the column; for the “green” cell extracts: 98:2:0% B (5 min); 48.5: 1.2:50, 0:0: 100 (3 min) followed by 6-min re-equilibration. Eluted component spectra were monitored in the range 400–700 nm. Free pigments were identified and quantified using authentic standards (Sigma, St. Louis, MS, USA). Astaxanthin esters were identified by their chromatographic mobility and quantified as free Ast.

### 3.5. Fatty Acid Analysis

Heptadecanoic acid (17:0) was added as an internal standard to the chloroform phase of the extracts obtained as specified above and the samples were transmethylated according to [38]. Methyl esters were extracted with *n*-hexane and immediately subjected to GC-MS analysis with an Agilent 7890 gas chromatograph equipped with DB-23 capillary column (Ser. No. US8897617H, 60 m × 0.25 mm, containing a grafted (50% cyanopropyl)-methylpolysiloxane polar liquid phase as a 0.25 μm-thick film) coupled with Agilent 5975С mass-selective detector (Agilent, Santa Clara, CA, USA). The fatty acid methyl esters (FAME) were separated under the following conditions: carrier gas (helium) pressure in the injector, 191 kPa; operational gas pressure in the column at 1 mL/min, carrier gas flow linear velocity in the column, 18 cm/s; sample volume, 1 μL; flow split ratio, 1:1; evaporator temperature, 260 °C. The oven temperature program was as follows: from 130 to 170 °C at 6.5 °C/min, to 215 °C at 2.75 °C/min (25 min at this temperature), to 240 °C at 40 °C/min, and 50 min at 240 °C, operational temperature of the mass selective detector, 240 °C, energy of the ionization, 70 eV. Identification of FA was done according to the retention times of standards (Sigma, St. Louis, MS, USA) and by characteristic mass spectra. The unsaturation index (UI) of FA mixtures was calculated as follows: UI = Σ p*_i_* × e*_i_*/100, where p*_i_* is the percentage and e*_i_*—the double bond number of *i*-th FA [38].

### 3.6. Microscopy

#### 3.6.1. Light and Luminescent Microscopy

The cells from the cultures were studied by bright field and fluorescence microscopy on Eclipse 90i (Nikon, Tokyo, Japan) motorized photomicroscope. For neutral lipid express assay, the cells were vitally stained with Nile Red fluorescent stain [39].

#### 3.6.2. Transmission Electron Microscopy

To prepare the samples for TEM, the cell suspension was washed twice with 0.1 M cacodylate buffer (pH 7.2) and fixed for transmission electron microscopic investigation according to a modified method suitable for the cells featuring a tough cell wall [40]. Ultrathin sections were prepared using an LKB 4800 ultramicrotome (LKB Produkter, Broma, Sweden), contrasted with lead citrate [41], and analyzed with a Hitachi HU-11F electron microscope (Hitachi Ltd., Tokyo, Japan) at an accelerating voltage of 80 kV.

#### 3.6.3. Scanning Electron Microscopy

For SEM sample preparation, the cells fixed and dehydrated in ethanol were transferred in absolute acetone and dried at a critical point in the HCP-2 dryer (Hitachi, Hitachi, Japan), coated with gold and palladium on by ion-sputtering IB Ion Coater (Eiko, Ibaraki, Japan) and examined in a JSM-6380LA (JEOL, Tokyo, Japan) microscope at an accelerating voltage of 15 kV.

## 4. Conclusions

The present work describes, to the best of our knowledge, the first strain of *H. pluvialis* isolated from an arctic seashore habitat characterized with moderate and highly variable salinity levels [42]. Indeed, the current compendia of White Sea algoflora do not include species from the genus *Haematococcus* other than freshwater representatives [43] which are not yet confirmed rigorously. The new strain was capable of massive accumulation of Ast as a secondary Car, mainly in the form of esters of the FA from the C16 and C18 families. Depending on the cultivation conditions, it turned out to be able to accumulate Ast in the amounts of 3%–5.5% of DW, which compares favorably with most of the currently known strains [2]. Our preliminary studies have shown that *H. pluvilais* BM1 seems to have an elevated salt tolerance, the extent of which, however, remains to be elucidated. Nevertheless, given its growth and biosynthetic capacity and potential salt tolerance, the new BM1 strain is a promising organism for biotechnological production of valuable carotenoids using microalgae. In particular, it merits close attention as a potential producer of Ast potentially capable of growth in brackish water.

## Supplementary Files

Supplementary File 1
