# Supplementary material for: Accumulation of Astaxanthin by a New Haematococcus pluvialis Strain BM1 from the White Sea Coastal Rocks (Russia)
Source: Mar Drugs. 2014 Aug 15;12(8):4504–20. doi: 10.3390/md12084504 (PMC4145328; doi:10.3390/md12084504)
Supplement: Supplementary File 1 [file marinedrugs-12-04504-s001.pdf]

# Supplementary Information

**Table S1.** Representative climatic parameters at the site of sample collection (summer, 2011).

| Month | Temperature, ℃ | | | | | | | Relative Humidity, % | | |
|---|---|---|---|---|---|---|---|---|---|---|
| | Air | | | Sea Water | | | Rock Bath | | | |
| | Avg. | Min | Max | Avg. | Min | Max | Max | Avg. | Min | Max |
| June | 13.5 | 4.5 | 30.5 | 10.0 | 4.4 | 14.7 | 31.5 | 69.2 | 24.5 | 95.5 |
| July | 16.1 | 6.6 | 25.7 | 14.5 | 8.5 | 16.9 | 40.2 | 75.2 | 35.1 | 96.4 |
| August | 12.7 | 6.0 | 23.4 | 14.5 | 10.4 | 16.8 | 29.6 | 76.8 | 34.6 | 95.6 |

**Figure S1.** Typical growth parameters of *H. pluvialis* BM1 "green" cell culture in the bubble column photobioreactor (see the Experimental section and). (**a**) Dry weight accumulation; (**b**) Changes in cell density; (**c**) Dynamics of total carotenoids and (**d**) chlorophylls.

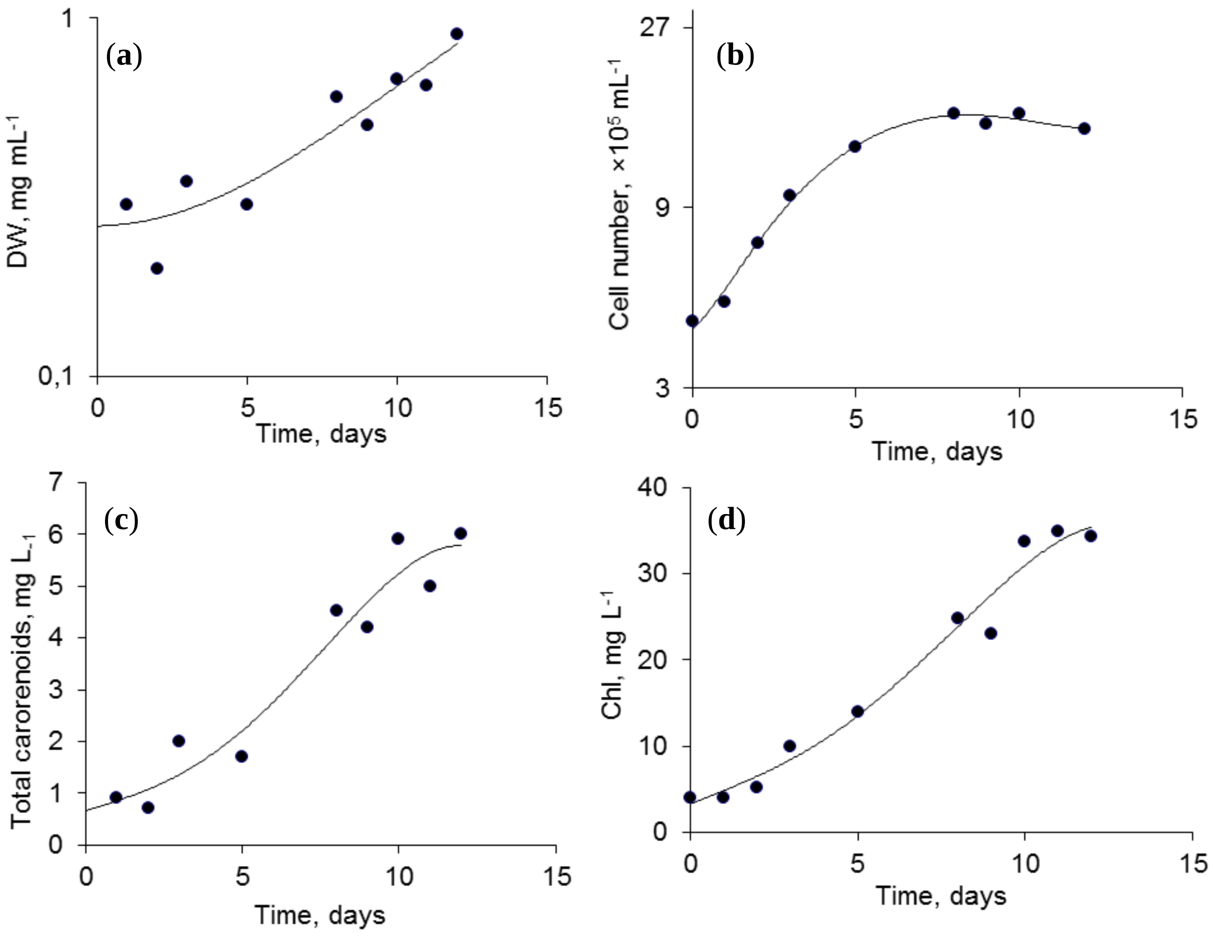

**Figure S2.** (**a**) The "red" cells of *H. pluvialis* BM1; (**b**) Absorbance spectra of the pigments extracted with dimethyl sulfoxide from the "green" cells and the "red" cells formed after a two-day stress exposition; (**c**) Kinetics of astaxanthin accumulation in *H. pluvialis* BM1 cells incubated under the stressful conditions (see the Experimental section); (**d**) The 'red' cells of *H. pluvialis* BM1 in the photobioreactor after a two-day stress exposition.

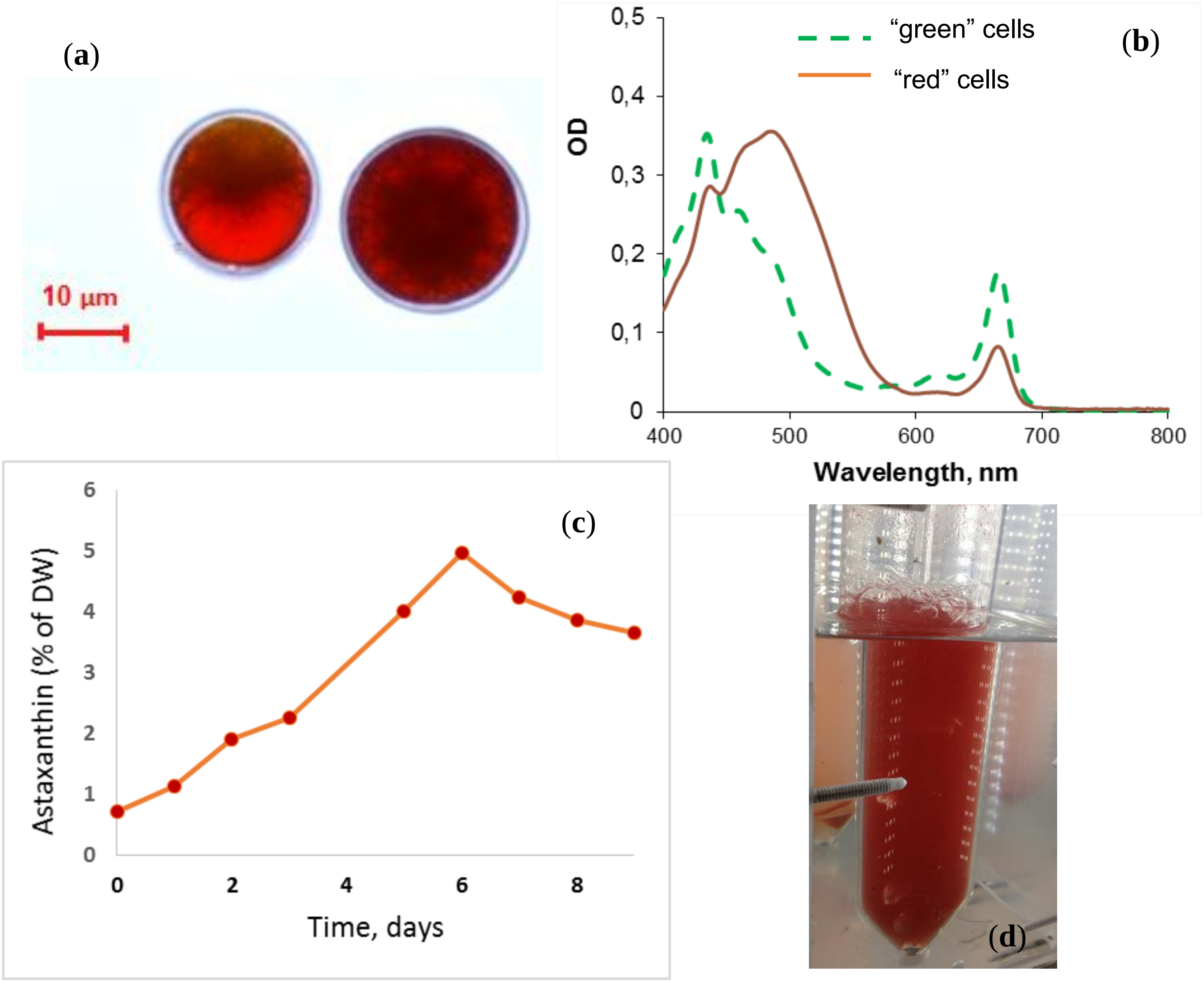


© 2014 by the authors; licensee MDPI, Basel, Switzerland. This article is an open access article distributed under the terms and conditions of the Creative Commons Attribution license (http://creativecommons.org/licenses/by/3.0/).
